# Radiofluorination using aluminum-fluoride (Al^18^F)

**DOI:** 10.1186/2191-219X-3-36

**Published:** 2013-05-08

**Authors:** William J McBride, Robert M Sharkey, David M Goldenberg

**Affiliations:** 1 Immunomedics, Inc., 300 The American Road, Morris Plains, NJ 07950, USA; 2 Garden State Cancer Center, Center for Molecular Medicine and Immunology, 300 The American Road, Morris Plains, NJ 07950, USA

**Keywords:** Radiofluorination, Fluorine-18, Peptides, PET, Molecular imaging, Review

## Abstract

Targeted agents are increasingly used for treating cancer and other diseases, but patients may need to be carefully selected to maximize the potential for therapeutic benefit. One way to select patients is to bind an imaging radionuclide to a targeting agent of interest, so that its uptake in specific sites of disease can be visualized by positron-emission tomography (PET) or single-photon emission computed tomography.

^18^F is the most commonly used radionuclide for PET imaging. Its half-life of approximately 2 h is suited for same-day imaging of many compounds that clear quickly from the body to allow visualization of uptake in the intended target. A significant impediment to its use, however, is the challenging coupling of ^18^F to a carbon atom of the targeting agent. Because fluorine binds to aluminum, we developed a procedure where the Al^18^F complex could be captured by a chelate, thereby greatly simplifying the way that imaging agents can be fluorinated for PET imaging. This article reviews our experience with this technology.

## Review

### Introduction

Molecular imaging with high-resolution positron emission tomography (PET) provides a sensitive and specific view of normal or abnormal biological processes or conditions that cannot be obtained through anatomical imaging. The most commonly used PET-imaging isotope is the halogen ^18^F. It has a highly abundant, low-energy positron emission (β^+^, 0.635 MeV (97%)), with a half-life of 109.8 min that provides the highest resolution of several common PET isotopes (^68^Ga, ^89^Zr, and ^124^I) [1]. It also has few undesired side emissions and is produced in a cyclotron from inexpensive and readily available ^18^O water, ^18^O(p,n)^18^F.

The best known fluorinated PET imaging agent is 2-[^18^F]fluoro-2-deoxyglucose ([^18^F]FDG), but there are increasing numbers of new imaging agents of potential medical interest. Naturally, ^18^F would not be the best choice for all targeting agents, but there are several receptors for peptides, such as integrins, somatostatin, bombesin/gastrin-releasing peptide, etc., that could be targeted by small peptides, where a radionuclide with a 2-h half-life, such as ^18^F, would be ideal [2].

^18^F usually is attached to the carbon atom of a prosthetic group and subsequently coupled to the targeting molecule [3-6], although attachments through silicon, phosphorus, and boron also have been employed [7-10]. The labeling of peptides with ^18^F on carbon is a multistep process, because harsh reaction conditions are used [6].

These methods typically start with ^18^F being trapped on an anion binding cartridge and then eluted with potassium carbonate and kryptofix-222. This solution is dried with heat under an inert gas and mixed with acetonitrile and dried again to remove the remaining water azeotropically, which reduces the nucleophilicity of the fluoride ion. The dry-down process can take 20 min with an automated set-up, but recently progress has been made to allow ^18^F to be attached in aqueous solution [11]. ^18^F is then used to displace a leaving group on the prosthetic molecule. The labeled prosthetic molecule is then purified by solid-phase extraction (SPE) or high performance liquid chromatography (HPLC).

The prosthetic molecule can then be attached to the targeting agent by many different methods, including oxime formation, acylation, alkylation, maleimide/thiol coupling, and click chemistry, to name a few [3-6]. The acylation and alkylation labeling methods are often used on small molecules, most likely with protecting groups present, so that only one reactive site is available in order to minimize side products. The oxime, maleimide/thiol, and click linkages can be used with more complex molecules, where the conjugation only occurs at specific sites. The maleimide method is often preferred for short-lived isotopes, because the reaction proceeds in minutes under very mild reaction conditions.

The ^18^F-prosthetic group is conjugated to the peptide or protein and then purified again. The entire labeling, purification, and formulation process often takes 1 to 3 h to perform, with decay-corrected yields often less than 40% [6]. The entire process, on a GMP manufacturing scale, typically takes 1 to 2 h, requires expensive automated equipment to produce the radiolabeled peptide. In addition, the complicated syntheses require a dedicated, highly skilled staff to produce the ^18^F-labeled molecules.

Unfortunately the process required to attach the ^18^F to a carbon atom on the targeting agent often is too long and cumbersome for practical use [3-6], which may hinder the development of new targeting agents of medical interest. Therefore, it would be a major advantage to have a simple, rapid method for binding ^18^F to a variety of compounds.

Our interest in developing a radiofluorinated peptide arose from studies with a bispecific antibody (bsMAb) pretargeting method that showed improved imaging capabilities over directly-radiolabeled antibody fragments [12]. This procedure utilized a radiolabeled hapten-peptide bearing a metal-binding chelate. Since ^18^F-metal complexes form quickly and in many instances very tightly [13], this provided the rationale to explore a peptide-chelate conjugate for rapid radiofluorination. In this review, we discuss the development of this technique and its potential for simplifying the preparation of ^18^F-labeled compounds for PET-imaging.

#### Aluminum fluoride complexes

Fluorine binds to most metals, forming a very strong bond with Al^3+^, which can form complexes with metal-binding chelates [13]. The aluminum fluoride bond is stronger than 60 other metal-fluoride bonds, e.g., bond energy of 670 kJ/mol [7,13]. The aluminum-fluoride bond is highly stable *in vivo*, and small amounts of AlF complexes are compatible with biological systems [14,15].

Perhaps, the biggest challenge at the onset was the selection of a suitable chelate that could hold the Al^18^F complex stably for several hours under physiological and biological conditions. Aluminum forms octahedral complexes; so ideally, a pentadentate ligand would be desired, leaving one binding site open for the fluoride ion. Naturally, the first ligands to examine would be those known to bind Al^3+^, with the caveat that (AlF)^2+^ was the actual material bound to the chelate. However, initially, studies began with a diethylenetriamine pentaacetic acid (DTPA) peptide, since DTPA was known to form a stable complex with another group III metal (^111^In) [16]. The test peptide, IMP272 (DTPA-QAK(HSG)Y_d_K(HSG)-NH_2_), included two hapten moieties (HSG is histamine-succinyl-glycine) on the lysine side chains for binding to the bsMAb used in pretargeting applications [17].

**Figure 1:**
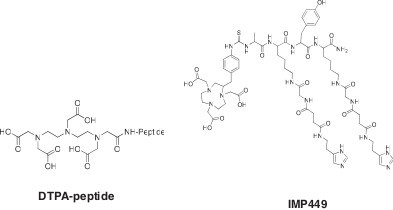
**Schematic structures of initial chelate-peptides used for Al**^**18 **^**F labeling.**

**Figure 2:**
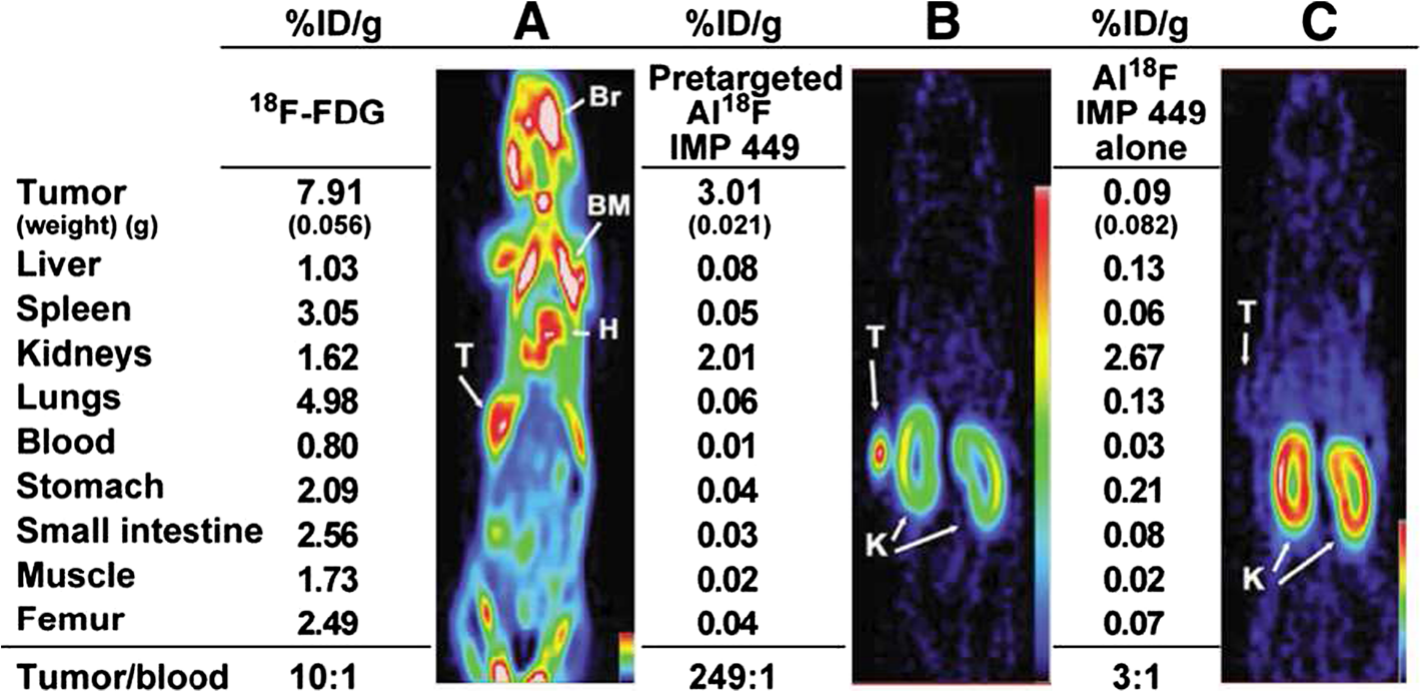
**Biodistribution of**^**18 **^**F-labeled agents in tumor-bearing nude mice by small-animal PET.** Coronal slices of three nude mice bearing small, subcutaneous LS174T tumor on each left flank after being injected with either (**A**) ^18^F-FDG, (**B**) Al^18^F-IMP449 pretargeted with anti-CEA x anti-HSG bsMAb, or (**C**) Al^18^F-IMP449 alone (not pretargeted with bsMAb). Biodistribution data expressed as percentage injected dose per gram (% ID/g) are given for tissues removed from animals at conclusion of the imaging session. Br, brain; BM, bone marrow; H, heart; K, kidney; T, tumor (reproduced with permission from the *Journal of Nuclear Medicine*; McBride et al. [21]).

The pH is critically important for the formation of (AlF)^2+^-chelate complexes. If the pH is too high, metals would form hydroxide complexes and precipitate, and if it is too low, then the preferred fluoride species in the equilibrium would be HF. Studies of AlF complexes suggested that pH 4 would favor a 1:1 aluminum-fluoride complex, and pH 4 was compatible with the metal-complex formation [18]. The ^18^F^−^, Al^3+^, and DTPA peptide were mixed together in a pH 4 buffer and heated, forming a complex with >90% yield, but it was unstable in water. Modifications to the peptide, adjacent to the DTPA, led to increased stability in water, but none was stable in serum [19]. The NOTA ligand was known to form stable complexes with Al^3+^[20], and thus, the commercially available S-2-(4-isothiocyanatobenzyl)-1,4,7-triazacyclononane-1,4,7-triacetic acid (*p*-SCN-Bn-NOTA) ligand was attached to a pretargeting peptide (IMP449, NOTA-p-Bn-CS-A_d_K_d_(HSG)Y_d_ K_d_(HSG)-NH_2_; Figure 1). The peptide was formulated in an acetate buffer (pH 4) and labeled with ^18^F^−^ by heating the mixture at 100°C for 15 min and then purifying by HPLC. The isolated labeling yield was low (5% to 20%), but the labeled product was stable in serum at 37°C (4 h); therefore, this product was used in preclinical testing using nude mice bearing the human colon cancer xenograft, LS174T [21]. Figure 2C shows a posterior coronal image taken 1 h after the animal was given Al^18^F-IMP449 (no pretargeting). Uptake was seen only in the cortical region of the kidneys (2.67% injected dose per gram (ID/g)). There was no bone accretion, and urine taken from the animals showed the labeled peptide was excreted intact, indicating stability *in vivo*. The center panel shows the animal given the Al^18^F-IMP449 following the bsMAb injection. In addition to the kidneys, the tumor (arrow identified with T) was clearly visible, illustrating the selective retention of the labeled peptide by the pretargeted bsMAb. The left panel shows an animal given the ^18^F-FDG, illustrating the high level of uptake in the brain, bone marrow, and heart (all sites active in metabolizing glucose), highlighting the improved visualization afforded by the bsMAb pretargeting method.

In a later study, Al^18^F-IMP449 was compared to a ^68^Ga-1,4,7,10-tetraazacyclododecane-1,4,7,10-tetraacetic acid-labeled peptide [22]. The targeting and biodistribution of the two peptides were quite similar, suggesting that the Al^18^F-complex was in a residualizing form of ^18^F, just like chelated radiometals, which are often sequestered within the cells that they target.

Efficient and stable binding of metals by chelates is highly influenced by the chelate structure. Thus, in an attempt to reveal how chelate structure influenced (AlF)^2+^ binding, we prepared three new pretargeting peptides, each with a different ligand. One had a 1,4,7-triazacyclononane-1,4-diacetate (NODA) ligand, and two had a NOTA derivative [23]. The four peptides (including IMP449) were labeled and purified by SPE using the same protocols. Table 1 shows that the simple NOTA ligand (IMP461) and the *p*-SCN-Bn-NOTA on IMP449 afforded roughly the same yield, while the NODA derivative on IMP460 had a much lower yield, possibly due to steric hindrance. All of the complexes formed with the peptides were stable in serum at 37°C. The IMP467 peptide contained the *C*-NETA ligand, which was known to have enhanced binding kinetics for some metals [24], and it did significantly improve radiolabeling yields. However, it formed two ^18^F complexes that could inter-convert (i.e., even when a single peak was isolated, in about 3 h at room temperature, it equilibrated back to the mixture). Importantly, the inter-conversion did not result in the loss of ^18^F from the complex. In contrast, the IMP460 and IMP461 peptides formed single complexes with (Al^18^F)^2+^.

**Table 1:** **NOTA/NODA ligands and maximum isolated yields after radiolabeling with 500 nmol peptide (*R*= K**_**d**_**(HSG)Y**_**d**_**K**_**d**_**(HSG)-NH**_**2**_**)**

**Peptide**	**Structure**	**Maximum**^**18**^**F-labeling yield (%)**
IMP449		44
IMP460		5.8
IMP461		31
IMP467		87

Reproduced with permission from Bioconjugate Chemistry, McBride et al. [23].

The one-step labeling of IMP467 was optimized further, completing the process within 30 min with only one SPE-purification step and a specific activity of 115 GBq/μmol (52% yield) [25]. Al^18^F-IMP467 also was stable *in vivo*, showing excellent targeting at 3 h with 8.16% ± 4.83%, 0.02% ± 0.01%, 0.41% ± 0.08% ID/g in the tumor, blood, and bone, respectively.

The ^18^F^−^ in the cyclotron target ^18^O water can contain metals, radiometals, and other impurities, so in most cases, it is purified before use. We also discovered that the readily available USP grade ^18^F^−^ in saline, a source of sterile and purified ^18^F^−^, could be used for the radiolabeling process. Using this product further simplifies the radiolabeling process and expands its use to radiopharmacies that do not have access to a cyclotron, thus affording widespread use of this new facile ^18^F-labeling kit.

**Figure 3:**
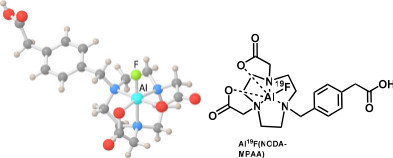
X-ray crystal structure of NODA-MPAA.

New simple NODA derivatives were synthesized by our group and also by Shetty et al., reporting an X-ray crystal structure of a NODA with a methylphenylacetic acid (MPAA) (Figure 3) or a benzyl group attached to the ring, respectively [25,26]. In both structures, the Al^3+^ forms a slightly distorted octahedral complex with the fluorine in an axial position. Labeling studies of various derivatives showed that having a carbonyl on the NODA ring or close enough to form a 5- or 6-member ring with the NODA reduced the labeling yield (11% to 24%). If a carbonyl group was 3 or 4 carbons removed from the ring, then the labeling yields were good (78% to 86%) [26]. These experiments indicate that the groups adjacent to the ligand can have a significant interaction with the complex. Furthermore, in cases where two peaks are seen for a NODA complex, the groups that are nearby may be hindering the free rotation of the AlF complex. The two isomers may be simply the complex with the ^18^F pointing in one direction relative to a nearby chiral center and pointing in the opposite direction for the other isomer. If the spacer is long enough, and/or does not have a functional group that interacts with the AlF complex, then a single peak is seen by HPLC.

The NODA-MPAA ligand was attached to a pretargeting peptide designated IMP485 (NODA-MPAA-K_d_(HSG)Y_d_K_d_(HSG)-NH_2_) [25]. Labeling yields were good, but adding a co-solvent in a 1:1 ratio to the aqueous radiolabeling solution of IMP485 significantly increased (e.g., doubled) the yield. Several different solvents, such as DMSO, DMF, CH_3_CN, and EtOH, also were effective, but EtOH was chosen because it was the most biocompatible of all the solvents.

#### Kit formulation

The goal was to make an IMP485-lyophilized kit that would contain most of the necessary components required for a successful, high-yield radiofluorinated product. The end-user would simply add USP ^18^F^−^ in saline and ethanol to the vial, heat for about 15 min and purify by SPE to obtain the final product within 30 min [27]. As with many compounds, having a suitable specific activity is critical. For example, the optimal specific activity for a somatostatin imaging peptide (approximately 28 GBq/μmol) has been examined, with lower uptake observed if the specific activity was too low or too high [28]. For pretargeting applications, we assumed that a specific activity of >18.5 GBq/μmol would be desired. We prepared a unit-dose kit that could be labeled at a cyclotron site or at a radiopharmacy some distance from a cyclotron, examining the amount of peptide, pH, radioprotectant, peptide-to-Al^3+^ ratio, bulking agent, and buffer needed to achieve a high-yielding product [27].

**Figure 4:**
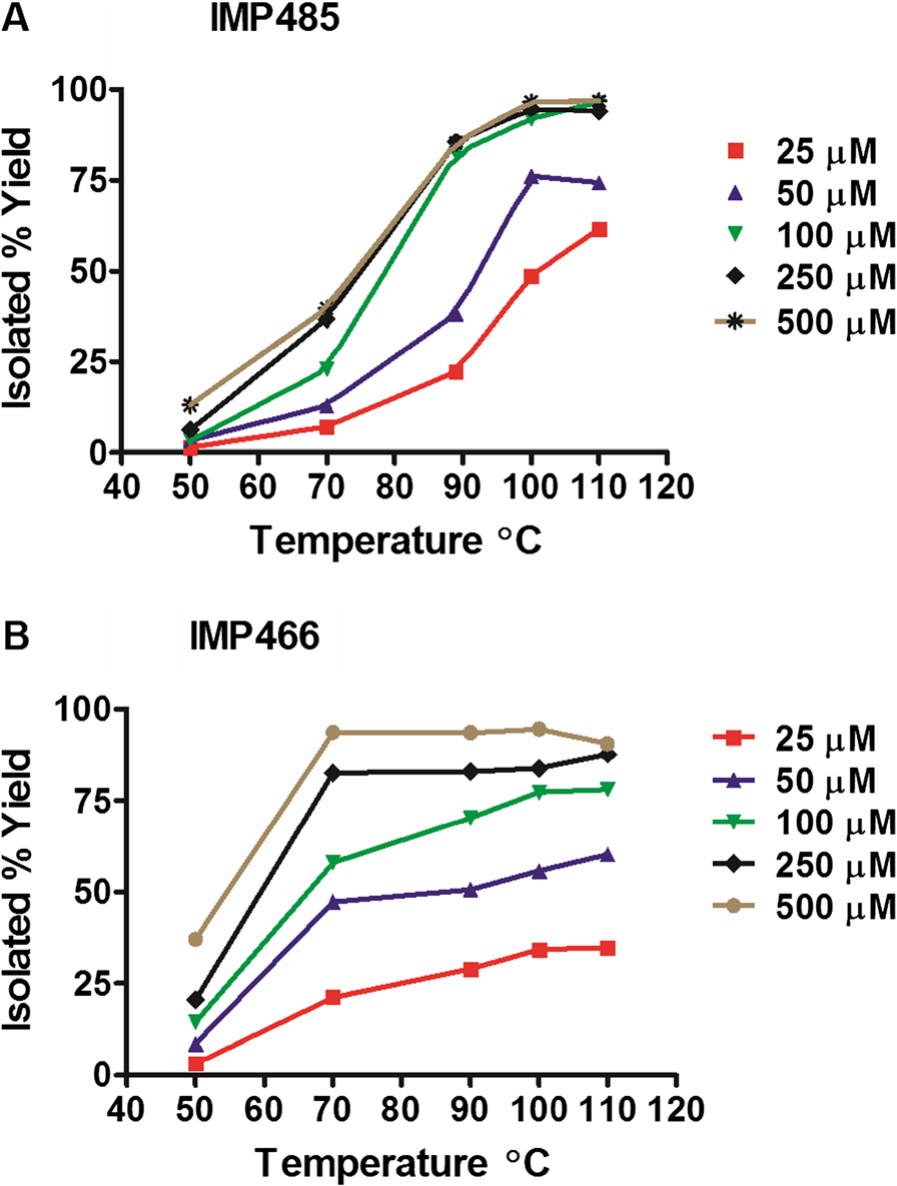
**Concentration of peptide in a kit versus radiolabeling yield heated 15 min at different temperatures.** (**A**) IMP485 and (**B**) IMP466 in 400 μL 1:1 saline:EtOH.

The optimum pH for radiolabeling was pH 4.0 ± 0.2, so two buffers (potassium biphthalate and ascorbic acid) were used to control the pH. The amount of the peptide chosen was 20 nmol, or a 50 μM concentration, when labeled in 400 μL of 1:1 Na^18^F in saline:EtOH. Under these conditions, the best yields of Al^18^F-IMP485 were acquired at 100°C to 110°C (Figure 4). A rapid SPE-purification step ensured > 97% purity. The kits were designed to provide a single-patient dose by adding approximately 1.85 GBq of Na^18^F to the vials. Under these conditions, yields typically ranged from 70% to 80%, with a specific activity of 92.5 GBq/μmol. When the final formulation was prepared using a GMP lyophilizer, radiolabeling yields improved to nearly 90%.

The highest specific activity IMP485 kit radiolabeling so far is 223 GBq/μmol, but the yield at this higher specific activity was just 45.6% compared to 80% to 90% when labeling at about 70 GBq/μmol. We also discovered that higher specific activity labeling requires additional attention to pH control.

#### Application of AlF to other agents

After establishing proof of principle with a pretargeting peptide, it was important to determine if this procedure would have broader utility with other compounds. The following sections summarize additional studies performed by our groups in collaboration with others, as well as other independent assessments of the procedure.

#### Receptor-targeting peptides

The same lyophilized kit formulation (20 nmol peptide, KHP/ascorbate buffers, pH 4.1, etc.) was applied to an octreotide analog, IMP466, NOTA-FCFW_d_KTCTol [27]. The peptide was labeled in exactly the same way, using 200 μL of ^18^F (2.51 GBq) in saline, with 200 μL ethanol added to the kit and heating to 100°C to 110°C for 15 min followed by purification by SPE. While the yields with this ligand (55%) were not quite as high as IMP485 with the MPAA-NODA ligand, the peptide could still be produced with a specific activity (60.5 GBq/μmol) that was suitable for *in vivo* imaging studies (Figure 4). The NOTA ligand on IMP466 had higher yields than that on IMP485 at lower temperatures, but IMP485 gave better yields at higher temperatures. This leads to the possibility that many different peptides or small molecules might be labeled and purified in a similar manner with subtle changes to achieve optimum yields.

IMP466 also was labeled in solution using a higher dose of peptide in a two-step, one-pot solution process that afforded the Al^18^F-IMP466 in 97% decay-corrected yield after HPLC purification [29,30]. The radiolabeling and tumor targeting of this peptide were confirmed by others [31]. Interestingly, this complex had two radiolabeled peaks by HPLC, while the same ligand on IMP461 (Table 1) formed a single Al^18^F complex. The two peaks are most likely due to the hindered rotation of the complex caused by an interaction of the complex with the sterically constrained cyclic peptide.

**Figure 5:**
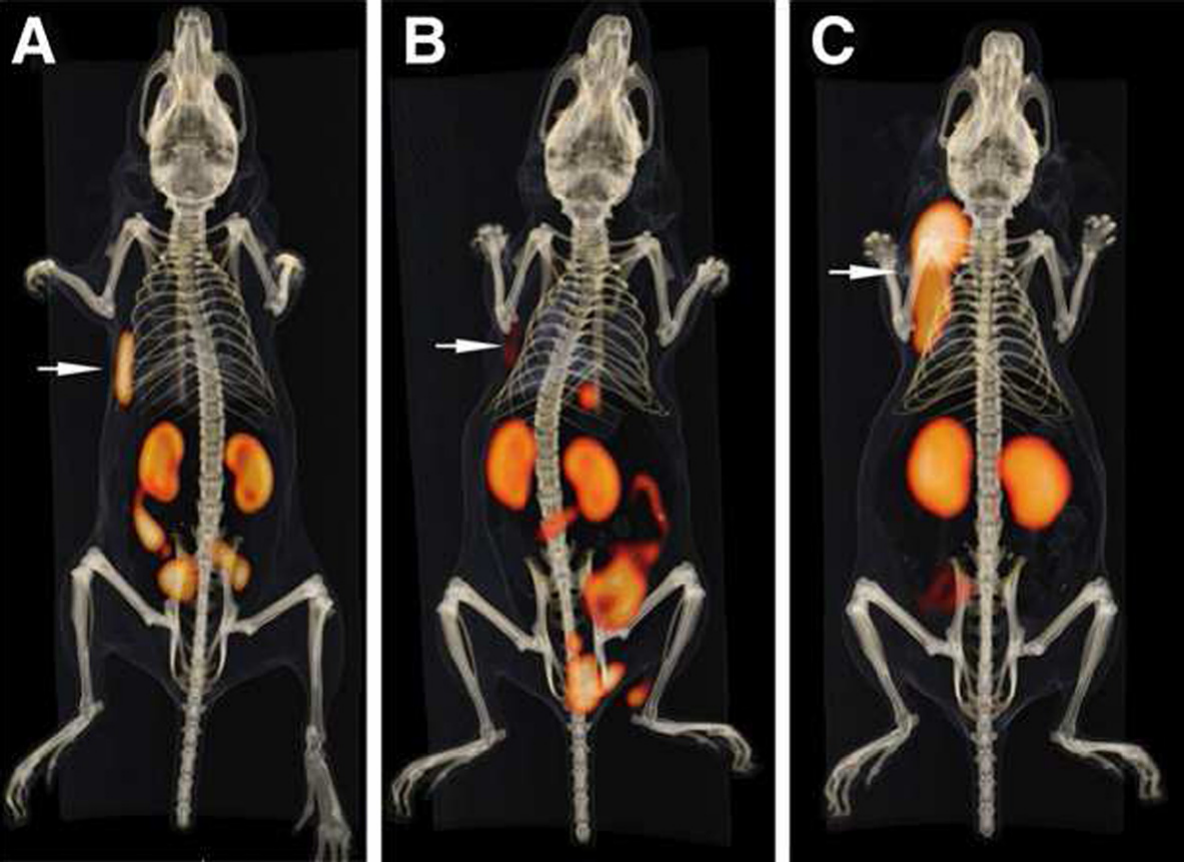
**Anterior 3D volume-rendered projections of fused PET and CT scans.** Mice with subcutaneous AR42J tumor on right flank injected with ^18^F-IMP466 (**A**), ^18^F-IMP466 in the presence of excess of unlabeled IMP466 (**B**), and ^68^Ga-IMP466 (**C**). Arrows indicate tumors. Scans were recorded at 2 h after injection. Reproduced with permission from the *Journal of Nuclear Medicine*; Laverman et al. [29].

The receptor targeting of the Al^18^F-NOTA-octreotide analog was compared to the same peptide labeled with ^68^Ga. Both peptides were stable *in vivo* and showed excellent and specific tumor targeting (Figure 5) in an AR42J rat pancreatic tumor model.

Dijkgraaf et al. [32] described the preparation and biodistribution of a bombesin peptide, NOTA-NH-(CH_2_)_7_CO-QWAVGHLM-NH_2_ (NOTA-8-Aoc-BBN(7–14)NH_2_) [33]. The peptide was radiolabeled in solution using ^18^F^−^ in saline, 80 nmol AlCl_3_, and approximately 80 nmol of the peptide in a pH 4.1 acetate buffer (100 μL aqueous total) and 400 μL acetonitrile. The solution was heated at 100°C for 15 min. The reaction solution was HPLC-purified to remove excess peptide and to remove a radiolytic impurity, which was expected for a methionine-containing peptide [34]. Radiolytic impurities also were observed with the thio-urea linked NOTA in IMP449 [21]. The reaction yield ranged from 50% to 90% with a specific activity of greater than 10 GBq/μmol after HPLC purification.

**Figure 6:**
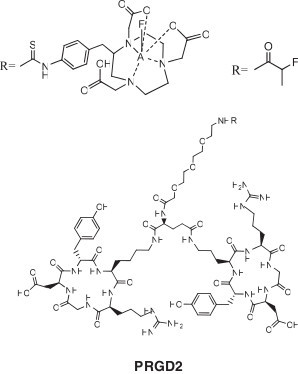
**RGD peptide of Lang et al. **[35]**.**

The arginine-glycine-aspartic acid (RGD) peptides are small cyclic integrin *α*_v_*β*_3_-targeting peptides used to localize sites of angiogenesis that can be used to image tumors, as well as damaged myocardial tissue [35-41]. The peptides were all labeled with ^18^F^−^ in solution and HPLC-purified. Four different AlF-binding ligands were used, the simple NOTA (same as IMP461, 45% yield) [36], a benzyl NODA (similar to NODA-MPAA, 58% yield) [38], the ITC-NOTA like IMP449 (5% to 42% yield) [35,41] (Figure 6), and the NODA-GA ligand of IMP460 (20% yield) [39].

Following our labeling method, Gao et al. [37] showed the Al^18^F-NOTA-PRGD2 produced a positive image of damaged myocardial tissue in contrast to the current heart imaging agents, ^201^Tl and (^99m^Tc) sestamibi, which provide negative images of damaged cardiac tissue.

A quantitative analysis of the Al^18^F-NOTA-PRGD2 was also performed in tumor-bearing nude mice that demonstrated that a [^68^Ga]Ga-NOTA-PRGD2 or [^18^F]FPPRGD2 had clearance patterns comparable for all three tracers [40]. The Al^18^F-NOTA-PRGD2 was tested recently in nine cancer patients, showing images of lung tumors, as well as illustrating renal excretion of this particular peptide without any noticeable uptake in normal tissues (e.g., bone) to suggest instability of the ^18^F [41]. This first-in-man experience confirms our initial assessment of the suitability of an Al^18^F-labeled product for *in vivo* use, as well as the simplicity afforded by this procedure using a lyophilized kit first reported by us [27].

#### Non-peptide, small molecule-imaging agents

**Figure 7:**
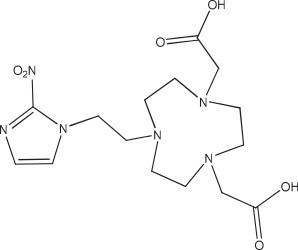
NODA-2-nitroimidazole.

**Figure 8:**
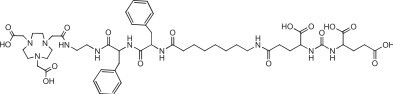
NOTA-DUPA-Pep.

A NODA-2-nitroimidazole derivative (50 nmol, 1 mL) (Figure 7) used for hypoxia imaging was labeled in 0.1 M, pH 4, NaOAc buffer by mixing with 22.5 μL of 2 mM AlCl_3_·6H_2_O (45 nmol) in 0.1 M pH 4 NaOAc, and 50 μL of ^18^F^−^ in saline, then heated at 110°C for 10 min to obtain the labeled complex in 85% yield [42]. *In vivo* studies with the Al^18^F-NODA-2-nitroimidazole showed the expected biodistribution and tumor targeting, with no evidence of product instability. The NOTA-DUPA-Pep molecule (Figure 8) was made for targeting the prostate-specific membrane antigen [43]. The ^18^F-labeled molecule was synthesized in 79% yield after HPLC purification to remove the unlabeled targeting agent.

#### Large peptide and protein labeling

NOTA-N-ethylmaleimide was attached to a cysteine side chain of the 40 amino acid exendin-4 peptide, which targets the glucagon-like peptide type-1 receptor [44]. The peptide was labeled with ^18^F^−^ using unpurified cyclotron target water to obtain the labeled peptide in 23.6% ± 2.4% uncorrected yield in 35 min. The Al^18^F-labeled peptide had 15.7% ± 1.4% ID/g in the tumor and 79.25% ± 6.20% ID/g in the kidneys at 30 min, with low uptake in all other tissues.

The NOTA-affibody Z_HER2:2395_ (58 amino acids, 7 kDa) was labeled at 90°C for 15 min with Al^18^F, with acetonitrile as a cosolvent [45]. The labeling and purification process took about 30 min, and the yield was 21% ± 5.7%. Again, biodistribution studies supported the stability of the product with negligible bone uptake.

**Figure 9:**
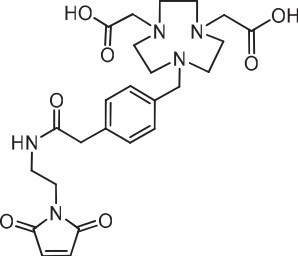
NODA-MPAEM.

We also examined a two-step labeling method for temperature-sensitive molecules [46]. The NODA-MPAA ligand was attached to N-ethylmaleimide to make NODA-MPAEM (Figure 9). The NODA-MPAEM (20 nmol in 10 μL 2 mM, pH 4, NaOAc) was mixed with 5 μL 2 mM AlCl_3_ in 2 mM, pH 4, NaOAc followed by 200 μL ^18^F^−^ in saline and 200 μL of acetonitrile. The solution was heated at 105°C to 109°C for 15 min and purified by SPE to produce the Al^18^F-NODA-MPAEM in 80% yield. This product was then coupled to a pre-reduced antibody Fab' fragment (20 nmol) by mixing the purified Al^18^F-NODA-MPAEM at room temperature for 10 min, followed by isolation of the labeled Fab' by gel filtration. The labeled protein was obtained in an 80% yield. The total synthesis time for both steps combined was about 50 min, with an overall decay-corrected yield of about 50% to 60%.

Several alternative two-step labeling methods also were explored, using azides/alkynes, aminoxy acetyl and thiols to link Al^18^F-NODA complexes to the complementary functionality on model compounds [47].

#### Residualization and *in vivo* clearance of Al^18^F complexes

Lang et al. compared the biodistribution of ^18^F on carbon, Al^18^F, and ^68^Ga attached to the same NOTA-PRGD2 (Figure 6) peptide in the U-87MG human glioblastoma model [35]. They found that the tumor uptake of the ^18^F-PPRGD2 peptide was 3.65% ± 0.51% ID/g at 30 min PI compared to 1.85% ± 0.30% ID/g at 2 h, indicating that the ^18^F activity was slowly clearing from the tumor between 30 min and 2 h (51% retention). The metal-complexed RGD peptides had higher tumor retention (4.20% ± 0.23% ID/g (30 min), 3.53% ± 0.45% ID/g (2 h) or 84% retention for Al^18^F-NOTA-PRGD2, and 3.25% ± 0.62% ID/g (30 min), 2.66% ± 0.32% ID/g (2 h), or 82% retention ^68^Ga-NOTA-PRGD2) over the same period. These data show that the chelated AlF complex may be retained better in the tumor than the radiofluorinated compound with ^18^F bound to a carbon atom. The retention of activity was also seen with the exendin peptide and the affibody, where the activity cleared from the kidneys when the ^18^F was attached to a carbon atom [48,49], but was retained with the Al^18^F complex [44,45]. Retention of the radionuclide in a tissue could provide a targeting advantage (e.g., [^18^F]FDG), particularly in rapidly metabolizing tissues, such as damaged heart tissue.

When designing an imaging agent for ^18^F, it is very important that the agent binds rapidly to the desired target and clears from normal tissues. The elimination and non-target tissue-binding properties of a given agent are defined by the structure of the radiolabeled molecule. If a molecule is predominantly lipophilic, it will have a high degree of hepatobiliary excretion; if it is hydrophilic, then renal excretion is more likely. In some cases, small lipophilic targeting molecules that have hepatobiliary excretion can be modified with negatively-charged groups and hydrophilic isotope-binding groups that can greatly reduce hepatobiliary excretion while increasing renal excretion of the non-targeted imaging agent [50-53]. With larger molecules, the clearance pattern will be determined mostly by the targeting molecule, but even there, occasionally small changes can have a pronounced impact on biodistribution [54].

## Conclusions

The Al^18^F labeling method is a versatile procedure that can be used with many targeting molecules (e.g., small molecules, peptides, and even proteins) that retain high binding affinities when derivatized with a NOTA ligand. A two-step labeling method can be used for temperature-sensitive molecules. The ligands and Al^18^F complexes are hydrophilic, which enables their use in aqueous systems. The labeling method is fast, simple, and can be accomplished in one or two steps in aqueous solution, which eliminates the need for a dry-down step needed for most ^18^F^−^ labeling methods. In some cases, molecules can be labeled in high yield and high specific activity, eliminating the need for HPLC purification; however, HPLC purification may be required in some circumstances. The labeling process is essentially the same from one compound to the next, requiring minimal efforts to optimize the method. The critical reaction conditions are pH (approximately pH 4), reaction temperature (100°C), concentration of reagents, and reaction time. The procedure is readily adaptable to automation on a simple, inexpensive, automated platform. Importantly, we showed the feasibility and practicality of having a lyophilized kit that can be simply taken off the shelf at any time and radiofluorinated in just 30 min. The Al^18^F-labeled molecules are stable *in vitro* and *in vivo*. The Al^18^F complexes are residualizing, which should provide an advantage for internalizing agents, while normal tissue retention (such as the kidneys) could potentially be minimized by slight modifications to the targeting molecule. The simplicity and adaptability of this procedure may expand our ability to introduce new molecular imaging agents in the future.

## Competing interest

WJM, RMS, and DMG are employed or have financial interest in Immunomedics, Inc.

## Authors' contributions

WJM, RMS, and DMG equally contributed to the making of this paper. All authors read and approved the final manuscript.

## Authors' information

WJM is the senior director of Peptide Chemistry at Immunomedics, Inc. He received his BS degree in Chemistry from U.C. Berkeley in 1979 and his PhD in Organic Chemistry from U.C. San Diego in 1984. From 1984 to 1985, he was a postdoctoral research associate at MIT in peptide synthesis. He joined Immunomedics, Inc., in 1994 and has specialized in making radiolabeled peptides for imaging and therapy. RMS holds PhD degree and is the senior director of Regulatory and Scientific Affairs at Immunomedics, Inc. After receiving his PhD at the University of Kentucky in 1982, he was involved in preclinical and clinical research, focusing primarily on radiolabeled antibodies, and more recently with bispecific antibody pretargeting. Prior to joining Immunomedics, he was a senior member and director of Clinical Research at the Center for Molecular Medicine and Immunology and its affiliated Garden State Cancer Center. DMG (ScD and MD) is the president and founder of the Center for Molecular Medicine and Immunology and its Garden State Cancer Center unit in Morris Plains, NJ. Beginning in 1972, when he transferred to the University of Kentucky Medical Center, he began developing models and preclinical targeting studies with radiolabeled antibodies to carcinoembryonic antigen (CEA), culminating in the first clinical studies on *radioimmunodetection* of cancer, and then later *radioimmunotherapy*, two terms he coined for antibody-based scintigraphy and targeted radiation therapy. His group has continued advancing these fields both preclinically and clinically using a number of cancer targets and studying naked antibody effects and mechanisms of action, as well as toxin and drug conjugates. He is also the founder of Immunomedics, Inc., and IBC Pharmaceuticals, Inc.
